# Validity of the Walked Distance Estimated by Wearable Devices in Stroke Individuals

**DOI:** 10.3390/s19112497

**Published:** 2019-05-31

**Authors:** Maxence Compagnat, Charles Sebiyo Batcho, Romain David, Nicolas Vuillerme, Jean Yves Salle, Jean Christophe Daviet, Stéphane Mandigout

**Affiliations:** 1 (Handicap, Aging, Autonomy, Environment) HAVAE EA6310, University of Limoges, 87042 Limoges Cedex, France; JYVES.SALLE@chu-limoges.fr (J.Y.S.); jean-christophe.daviet@unilim.fr (J.C.D.); mandigout@unilim.fr (S.M.); 2 Department of Physical Medicine and Rehabilitation, University Hospital Center of Limoges, 87042 Limoges Cedex, France; Romain-david@hotmail.fr; 3 Center for Interdisciplinary Research in Rehabilitation and Social Integration (CIRRIS), Centre Intégré Universitaire de Santé et de Services Sociaux de la Capitale Nationale (CIUSSS-CN), G1M 2S8 Quebec, QC, Canada; charles.batcho@fmed.ulaval.ca; 4 Department of Rehabilitation, Faculty of Medicine, Université Laval, G1M 2S8 Quebec, QC, Canada; 5 AGEIS, University Grenoble Alpes, Grenoble, 38 041 Grenoble CEDEX 9, France; nicolas.vuillerme@univ-grenoble-alpes.fr

**Keywords:** accelerometry, wearable electronic devices, validity, walking, physical activity

## Abstract

Background: Health professionals need valid devices to assess a stroke individual’s ability to walk. The aim was to evaluate the validity of the estimation of the walked distance by wearable devices and the impact of the sensor’s position in stroke individuals. Methods: Post-stroke patients able to walk without human assistance were equipped with several wearable devices: pedometers, Actigraph, and Sensewear Armband placed according to the manufacturers' recommendations. Participants walked for 6 min at a comfortable speed wearing all sensors at the same time. We analyzed the validity of sensor-estimated distances according to their position using Bland–Altman analysis, root-mean-square error, and coefficient of correlation. Results: In total, 35 individuals were included (mean age = 65 ± 15 years). The best estimations were given by the Actigraph worn on the unaffected ankle (mean bias (MB) = 22.6 ± 32.4 m; *p* = 0.37) and by the pedometer worn on the unaffected hip (MB = 20.5 ± 24.6 m; *p* = 0.46). The other sensors and positions provided large estimation errors over 95 m (*p* < 0.05). Conclusion: This study led to a recommendation of a pedometer worn on the unaffected hip or an Actigraph worn on the unaffected ankle to get a valid estimation of the distance walked by stroke individuals.

## 1. Introduction

Walking is a key activity to improve the quality of life and social participation in individuals with stroke sequelae [1,2]. However, studies showed that 30% of patients do not recover the ability to walk independently six months after the stroke, and nearly 65% consider that their social participation is limited [3]. The most often reported parameters in the literature to estimate an individual’s capacity to walk in a social environment are the speed—commonly evaluated with a 10-m test—and the distance (d), assessed with a 6-min walk test [4]. Perry et al. demonstrated that a speed over 0.4 ms^−1^ was the threshold defining the ability to walk in a social environment [5]. However, there may be a gap between a subject’s performance in these tests and the actual capacity to walk freely due to obstacles in the environment [6,7]. This is especially true since the distances walked in social situations are significantly longer than in tests [8]. For example, Salbach et al. showed in a study including 24 cities around the world that an individual has to travel a mean distance between 57 and 98 m to go to a post office, between 77 m and 114 m to a place of worship, 260 m for a doctor appointment (SD: 78 m), and 129–381 m to go to a shopping mall [4]. 

To assess the individual’s real walking ability outside of institutions, health professionals need reliable and practical tools that can be used in daily life situations [7,9]. The most commonly used tools to measure the walked capacity in terms of distance or number of steps are global positioning systems (GPSs), pedometers, and accelerometers [10]. On the one hand, the number of steps tends to be an unreliable parameter for medical practitioners as the step length is variable depending on the type and level of motor deficiency, which causes a lower comparability between individuals [11,12]. On the other hand, distance is more reliable, with better comparability between individuals and readily accessible data pertaining to social participation in the literature [2,4,8,13]. It can be measured accurately using a GPS, but its use is limited to outdoor situations due to the necessity of satellite synchronization [14].

Accelerometers and pedometers can be used indoors and were validated for estimations of the walked distance in healthy populations [15,16]. These systems generally use the product of the step count and the step length. Step length is evaluated by measuring the distance traveled over 20 steps [15]. However, this method remains debatable in post-stroke subjects because of the variability of a given individual’s step length [17]. Furthermore, Crouter et al. reported that the accuracy of these devices sharply drops when individuals walk slower than 0.8 ms^−1^ [15], and post-stroke patients typically tend to walk slower than healthy individuals [18]. Caroll et al. indeed demonstrated in a population of 51 post-stroke individuals that pedometers placed on the hip or around the neck were unable to detect steps when walking slower than 0.5 ms^−1^ [19]. Other types of sensors are, however, able to make accurate estimations of the number of steps even in slow-walking individuals. For instance, Fulk et al. demonstrated that the accelerometer-based step activity monitor had an estimation error of five steps in a 2-min walk test with an intraclass coefficient correlation (ICC) of 0.97, even in subjects walking slower than 0.5 ms^−1^ [20]. Additionally, Klassen et al. demonstrated that the location of the sensor had a direct impact on the results; a triaxial accelerometer reported a standard error lower than 15% when placed on the ankle, while its standard error was greater than 80% when placed on the hip in a study including 43 post-stroke subjects. The impact of the sensor’s position was even greater in individuals who walked slowly (<0.5 ms^−1^) [21]. 

Naturally, when confronted with a large number of possible devices and positions recommended by the manufacturer, medical practitioners and users are at a loss when it comes to choosing the right sensor and position to obtain the best estimation of walked distance. Therefore, it seems essential to check the validity of each option to point toward a global recommendation in post-stroke populations. 

The objective of this study was to evaluate the validity of the walked distance estimations by pedometers and accelerometers and to evaluate the impact of their position (ankle, hip, wrist/affected, unaffected side) in a post-stroke population walking at a comfortable speed.

## 2. Materials and Methods

### 2.1. Participants Selection

Participants were recruited in the Physical Medicine and Rehabilitation Department. The inclusion criteria were (1) a single stroke confirmed with brain imaging, and (2) the ability to walk continuously for 6 min without human assistance. Participants with acute cardiac or respiratory pathologies or decompensated chronic pathologies were excluded. 

The health professional responsible for the protocol informed the patients of the details of the protocol and registered their consent. The research protocol was accepted by an ethics committee, notice number CERNI 2015-01-13-57.

### 2.2. Assessment of the Hemiplegia 

Motor function was evaluated using the Demeurisse motricity index [22]. Spasticity was evaluated using the modified Ashworth scale (MAS) [23]. Walking autonomy was assessed using the functional ambulation classification modified (FACm) [24]. Autonomy related to activities of daily living was evaluated using the Barthel index [25]. All these evaluations were performed by the same examiner for all participating subjects.

### 2.3. Instrumentation

We selected several accelerometers with various technological features which were previously used in several studies on physical activity in post-stroke populations [26,27,28]. This selection was based on the type of device (pedometer [19], accelerometer [29], multisensor [30]) and the position options [31] (additional information is provided in Table S1).

#### 2.3.1. Actigraph GT3x

The Actigraph GT3x (Actigraph LLC, Pensacola, FL, USA) is the most widely used accelerometer for physical activity evaluations in clinical research [32]. The Actigraph GT3x is a small (4.6 cm × 3.3 cm × 1.5 cm) and lightweight (42.5 g) triaxial accelerometer designed to measure accelerations in the range of 0.05–2 *g* with a band-limited frequency of 0.25–2.50 Hz. This corresponds to the range in which most human activities are performed [33]. We chose a standard configuration with a standard sampling frequency of 30 Hz and no specific filtering. The device was initialized using 1-s epochs. The Actigraph data can be downloaded to a personal computer via a reader interface unit. In our work, we used the step count estimate provided by the ActigraphGT3x. It can be worn on the wrist, hip, or ankle [34]. We chose to place the devices on the wrists and ankles on the affected and unaffected sides, and on the hip on the unaffected side. The sensor was only placed on the unaffected hip because Rand et al. [35] reported that there was no difference in the estimation of the number of steps whether the sensor was on the affected or unaffected hip. This was, therefore, done to simplify the protocol. The sensor was attached to the different placements by an elastic band provided by the manufacturer. This choice was made to judge the impact of the impairments and the positioning of the estimations of the sensor. 

#### 2.3.2. Sensewear Armband

A multisensor array (Sensewear Armband, Body Media, Pittsburgh, PA, USA) was positioned on the back of the participant’s arm, midway between the shoulder and elbow. It measures 7 cm × 7 cm × 2 cm and weighs 55 g. It gathers raw physiological data of movements using a biaxial accelerometer and records heat flux, skin temperature, and galvanic skin response. Cycles of 1 s were used in this study, i.e., accelerations were integrated over 1-s periods to produce a number of counts for each second, which was written into the internal memory. No specific filtering was used. At the end of each trial, the stored Sensewear Armband data were downloaded to a personal computer for analysis using the reader interface unit supplied by the manufacturer. We used the step count estimate provided by Sensewear Armband for our work. We chose to place the devices on the affected and unaffected arm to judge the impact of the impairments of the estimations of the sensor. The sensor was attached to the arm by an elastic band provided by the manufacturer.

#### 2.3.3. Pedometer (ONStep 400, Geonaute)

The ONStep 400 (Decathlon France S.A.S, Villeneuve d’Ascq, France) is an inexpensive pedometer available to the general public. Its size is 6.5 cm × 4.1 cm × 1.5 cm, and its weight is 40 g. It can be worn on the hip, in the pockets of trousers, or around the neck using a strap [36]. The data recorded by the pedometer can be observed directly on the device, which does not require specific software. The recording is triggered when the subject initiates the first step and ends when the subject is no longer in motion. This device uses a piezoelectric mechanism to count the number of steps. We used the step count estimate provided by the pedometer for our work. We chose to place the devices on the unaffected hip and around the neck to judge the impact of the placement on the estimations of the sensor. The sensor was attached to the limb by an elastic band for hip placement and a fabric strap provided by the manufacturer for placement around the neck.

### 2.4. Walked Distance

The participants were required to walk at a comfortable speed on a circuit with no half-turn or obstacle, graduated every 5 m. The distance walked by each patient was measured by the examiner using the scales marked on the ground. The estimated walked distance was calculated by multiplying the number of steps reported by the wearable devices by the average step length. The average step length was calculated using the method described by Bassett and Crouter [15,16]. This method consists of asking the participant to walk 20 steps at a comfortable speed and then measuring the distance walked. The average step length was then obtained by dividing this distance by 20. To weigh the variability of this measurement, we asked the subject to repeat the operation three times, after which we calculated the average step length from the three trials.

### 2.5. Test Protocol

**Figure 1:**
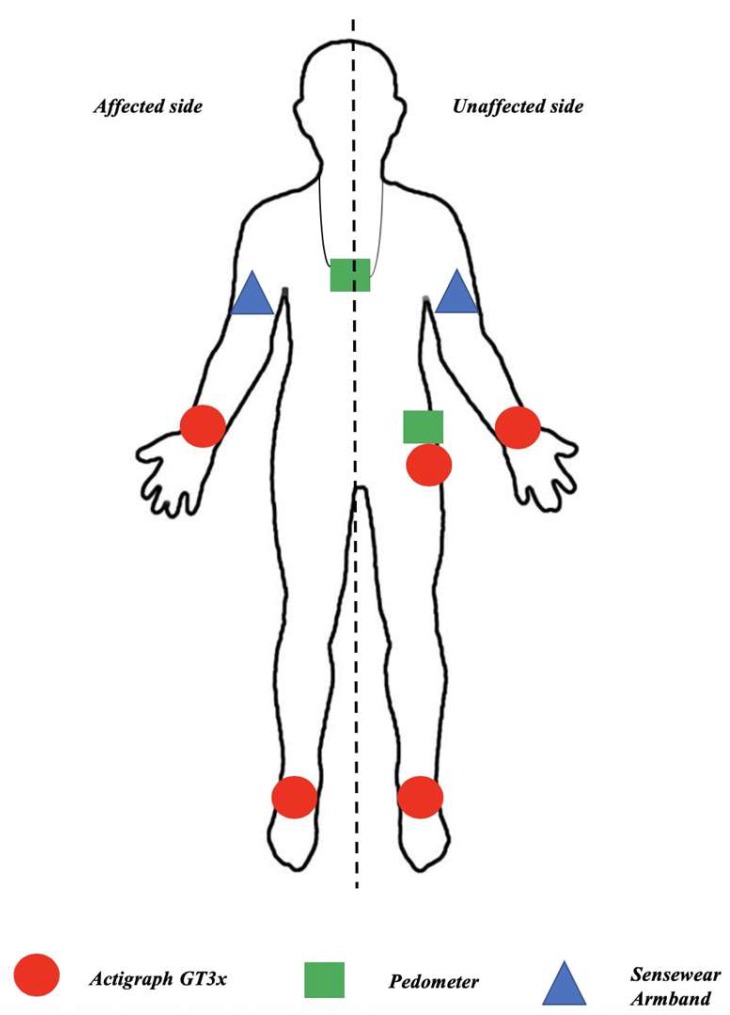
Placement of the devices.

The test protocol involved the following steps:

1. Measurement of the average step length over three trials of 20 steps.
2. Installation of the sensors. Actigraph GT3x devices were placed on the wrists and ankles on both the affected and unaffected sides, as well as at the unaffected hip. Sensewear Armbands were placed on both the affected and unaffected arms. Pedometers were placed at the unaffected hip and around the neck. The device placements are illustrated in Figure 1.
3. The participants performed a six-minute walk test at a comfortable walking speed. During this walking period, the distance walked was measured by the examiner with the graduations marked on the floor of the corridor.
4. Download of the data from all devices.

### 2.6. Statistical Analysis

Based on the work of Carroll et al., we calculated the necessary sample size by predicting a mean difference of 10% and a standard deviation of 20% between the estimated and measured distances [19]. Thus, from a Bland–Altman analysis, we planned to include a total of 32 subjects in order to reach 80% power with an alpha-risk of 0.05 [37]. The validity of the walked distance estimated by wearable devices was determined by analyzing the accuracy, and the agreement for each device in comparison to the criterion measure. These were defined by the mean bias (MB) and 95% limits of agreement (95% LoA) on the Bland–Altman analysis, root-mean-square error (RMSE), Pearson’s correlation coefficient (*r*), and coefficient of determination. We performed a paired-sample Wilcoxon test to analyze the significance of the difference between the measured distance and the distance estimated by each sensor according to their position. The threshold of significance was 0.05. All calculations were performed using the RealStats 2011 software (Real Statistics Using Excel© 2012–2019, Charles Zaiontz).

## 3. Results

### 3.1. Population Characteristics

We included 35 subjects with a mean age of 64.6 ± 14.8 years. The participants showed heterogeneous levels of deficiency and autonomy (see Table 1). The mean step length was 0.46 ± 0.11 m.

### 3.2. Validity of the Analysis

Across all recording devices, the Actigraph data of one patient were lost and six armband devices (17% of armbands) had a recording failure. The reasons for these recording issues were not found. 

**Figure 2:**
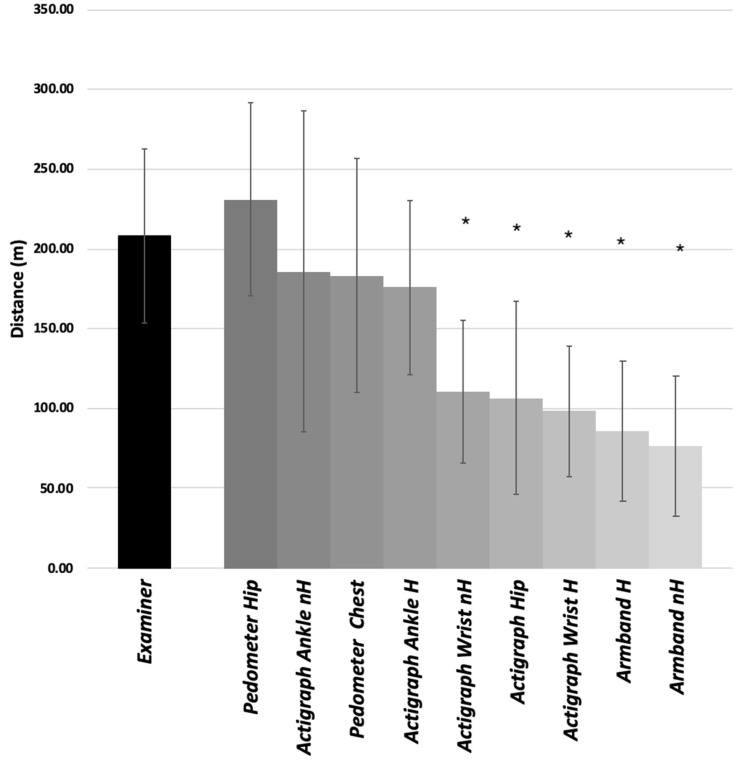
Distance measured by the practitioner and estimated from step counts reported by all devices according to type and placement. nH: non hemiparetic side; H: hemiparetic side. * *p* < 0.05 at the end of the Wilcoxon test comparing the distance measured by the examiner and that estimated by the device from the number of steps.

The mean walked distance measured by the examiner at the end of the test was 208.2 ± 109.2 m. No significant differences were found between the distance measured by the examiner and the following devices: the Actigraph worn on the unaffected ankle (d = 185.7 ± 100.6 m; *p* = 0.37), the Actigraph worn on the affected ankle (d = 175.7 m ± 109.3 m; *p* = 0.21), the pedometer worn around the neck (d = 183.4 m ± 147.2 m; *p* = 0.32), and the pedometer worn at the non-affected hip (d = 231.1 m ± 121.2 m; *p* = 0.46). For all other combinations of sensors and locations, there was a significant measurement error with *p* < 0.001 (Figure 2). The step count of each device is illustrated in Table 2.

### 3.3. Validity Parameters

The parameters of validity of each sensor are summarized in Table 3. The most accurate estimations were obtained using the pedometer worn at the hip on the non-affected side (MB = 9.7%, RMSE = 10.9%) and the Actigraph placed at the ankle on the non-affected side (MB = 10.7%, RMSE = 14.6%). On the other hand, the pedometer worn on the hip and the Actigraph worn on the ankle on the affected side showed the best coefficients of correlation (*r* > 0.90) and the lowest limits of agreement. The Actigraph worn on the ankle on the unaffected side had lower correlation (*r* = 0.93) and higher 95% LoA (111.4; −46.4 m) compared to the same device located on the affected limb, even though no statistical difference was observed between the two estimations. 

## 4. Discussion

The objective of this work was to evaluate the validity of the estimations of walked distance by wearable devices in individuals with stroke sequelae. We observed that the best estimations over a 6-min walk at comfortable speed were provided by the pedometer worn at the hip on the non-affected side (MB = 9.7%, RMSE = 10.9%) and by the Actigraph worn at the ankle on the non-affected side (MB = 10.7%, RMSE = 14.6%). 

We observed significant differences between the combinations of sensor type and position, which demonstrates the impact of these parameters. For instance, despite being placed in the same location (hip on the non-affected side), the pedometer provided a better estimation of the walked distance than the Actigraph, even though the first is a piezoelectric device and the second is a triaxial accelerometer. On the one hand, the Actigraph’s measurement error was considerable (MB = 101.8 ± 60.1 m; RMSE = 60.3 m) compared to the pedometer (MB = 20.5 ± 24.6 m; RMSE = 23.1 m). This could potentially be explained by an issue in the settings of the Actigraph or in its algorithm. The chosen settings were standard, i.e., a standard sampling frequency (30 Hz) and no specific filtering. It is possible that the use of other algorithms or other settings may alter the accuracy of the Actigraph GT3X. The manufacturer of this device recently published an add-on called “low-frequency filter extension” which can be enabled for healthy individuals with low amounts of physical activity [34]. This add-on lowers the detection threshold of the Actigraph to improve the data acquisition sensitivity [34,38]. It would be relevant to evaluate the impact of these settings on the estimation of the subject’s walked distance, especially in individuals with limited walking capabilities. 

On the other hand, the pedometer might have a lower detection threshold, as it is specifically designed to count the number of steps of an individual. This would, therefore, induce a better sensitivity in this population. However, we are unable to confirm these hypotheses since we could not analyze the raw data used by the sensors. Like the Actigraph, the Sensewear Armband had a consequential measurement error despite being a multisensor validated for the assessment of energy expenditure in post-stroke populations, with an MB of 127.3 ± 79.8 m and RMSE = 65 m when placed on the affected side, and an MB of 120.6 m ± 83.8 m and RMSE = 80 m when placed on the unaffected side. These results are consistent with those reported by Manns et al., who observed an error of 193.1 ± 168.1 steps in two six-minute walk tests in a sample of 12 post-stroke subjects [30]. Similar results were also reported by Vanroy et al., whose study reported an estimation error between 110 and 190 steps using the Sensewear Armband after walking 120 m at a comfortable speed in a group of 14 post-stroke subjects [39]. Thus, this device does not seem reliable to estimate the number of steps in post-stroke subjects. The cause may be the device’s algorithm, which might fail to correctly count the number of steps, but the impossibility of accessing the raw data prevented us from confirming this hypothesis. In post-stroke populations, we would recommend limiting the use of this sensor to its main function, i.e., assessing the energy expenditure. 

The sensor’s position on the body also had a significant impact, as proven by the differences in the results of the Actigraph between the ankle, hip, and wrist. The estimation of the Actigraph worn at the ankle was the closest to the measured walked distance (MB = 22.6 ± 32.4 m), while the same device worn at the hip or on the wrist had MB values over 95 m (Table 3). It is possible that placing the Actigraph on the ankle provides a better exposure to the accelerations of the limb, which would enable a better acquisition of the number of steps. This hypothesis is supported by Klassen et al., who demonstrated that placing a Fitbit triaxial accelerometer on the ankle provided a lower estimation error, from 84.6% ± 30.5% when placed at the waist to 15.8% ± 22.3% when placed on the ankle in a study including 43 post-stroke subjects [21]. 

### 4.1. Strengths of the Study

This study brings light on the precision of several types of sensors and the impact of their position on the estimation of the distance walked by individuals with stroke sequelae. Overall, the devices had an estimation error of about 100 m after walking for 6 min, with the exception of the pedometer and the Actigraphs worn on the ankles, which provided better results. Knowing that stroke survivors walk at an average speed of 0.5 ms^−1^ (1.8 kmh^−1^), this estimation error would cause an underestimation of about 1 km per hour of walking, which would amount to more than 50% of the measured distance. The most accurate estimation was obtained using the Actigraph on the ankle on the unaffected side and the pedometer on the hip on the unaffected side with a mean standard error of about 20 m after a 6-min walk, which corresponds to 200 m per hour of walking. Therefore, according to these results, medical practitioners should use either the Actigraph placed on the unaffected ankle or the pedometer placed at the hip. Between these two choices, the pedometer appears to be an attractive solution due to its low price. Additionally, our results bring to both the practitioner’s and the end-user’s attention the accuracy issues of these devices and the necessity to wisely choose the position and type of the sensor to avoid unreliable estimations. 

### 4.2. Limitations

We are aware of two limitations of our study. The first limitation is the method used to estimate the walked distance. We used the average step length, which can be variable in one individual during a single activity or along the day, especially in the stroke population [11,17]. The sensor was set on a constant step length, which might have induced an overestimation of the distance if it counted one full step while the subject was limping. It may be necessary to analyze the accuracy of these devices in a real-life situation over several days at home to get more precise data. Even so, the technique we selected to estimate the walked distance using the subject’s average step length enabled us to make an initial configuration of these devices for a population of post-stroke subjects, which seems critical to improve the use of these tools in a clinical setting. The second limitation of our study pertains to the external validity of the results. A plethora of physical activity trackers exist, and many of them could have been included in the study (e.g., step activity monitor, smartphones, smartwatches, etc.). We intended to use the three most commonly used technologies: piezoelectric devices (pedometers), triaxial accelerometers, and multisensors [32]. However, our results may be difficult to expand to other untested devices due to the possible impact of their specific algorithms on the estimated number of steps. 

## 5. Conclusions

The validity of the estimations of the walked distance by wearable sensors varied significantly according to the device’s type and location. Following our results, we recommend using a pedometer (piezoelectric device) worn on the hip on the unaffected side, or the Actigraph activity monitor (triaxial accelerometer) worn on the ankle on the unaffected side to estimate the walked distance in individuals with neurological sequelae of stroke.

- The sensor type and its location on the body strongly impact the estimation of the walked distance in individuals with stroke sequelae.
- The pedometer (piezoelectric device) placed on the hip and the Actigraph activity monitor (triaxial accelerometer) worn on the hip on the non-affected side provided the closest estimations of the walked distance.
- Placing an Actigraph on the upper limbs caused a significant underestimation of the walked distance in individuals with stroke sequelae.
- The Sensewear Armband strongly underestimated the walking distance regardless of its placement on the affected or unaffected upper limb of the stroke individuals.

## Figures and Tables

**Table 1:** Characteristics of the population. SD: standard deviation; BMI: body mass index; FACm: functional ambulation classification modified; MAS: modified Ashworth scale.

	MEAN / MEDIAN	SD	MIN	MAX
AGE (YEAR)	64.60	14.80	34	88
BMI (KG∙M^−2^)	26.70	5.50	20	43
TIME AFTER STROKE (DAYS)	781	1492	9	5110
DEMEURISSE UPPER LIMB SCORE (/100)	68		1	100
DEMEURISSE LOWER LIMB SCORE (/100)	77		43	100
MAS (/5)	1		0	4
BARTHEL INDEX (/100)	74		40	100
FACM (/8)	5		4	8
SPEED (MS^−1^)	0.56	0.30	0.06	1.22

**Table 2:** Step count reported by the devices. nH: non hemiparetic side; H: hemiparetic side.

	Pedometer Hip	Actigraph Ankle nH	Pedometer Chest	Actigraph Ankle H	Actigraph Wrist nH	Actigraph Hip	Actigraph Wrist H	Armband H	Armband nH
Mean step count (step)	514	410	406	387	237	221	212	195	170
SD step count (step)	251	188	295	216	166	235	161	249	196

**Table 3:** Validity parameters of distance estimated by wearable devices versus distance measured by examiner. Unit in meters; nH: non hemiparetic side; H: hemiparetic side; percentage difference: mean bias expressed in percentage of distance measured and estimated by device; 95% LoA: limits of agreement of Bland–Altman analysis; *r* = Pearson correlation coefficient; *p* = statistical significance of Pearson correlation coefficient; RMSE: root-mean-square error; percentage RMSE: RMSE expressed in percentage of distance measured by examiner.

	Mean Bias (m)	Percentage Difference (%)	95% LoA Up (m)	95% LoA Down (m)	Percentage 95%LoA (%)	*r*	*p*	RMSE (m)	Percentage RMSE (%)
Distance Actigraph Ankle nH	22.58	10.70%	87.45	−42.29	30.80%	0.95	<0.001	30.79	14.60%
Distance Actigraph Ankle H	32.50	15.40%	111.39	−46.38	37.40%	0.93	<0.001	40.20	19.00%
Distance Actigraph Hip	101.78	48.30%	222.37	−18.81	57.20%	0.86	<0.001	62.28	29.50%
Distance Actigraph Wrist nH	97.55	46.30%	228.04	−32.93	61.90%	0.79	<0.001	55.08	26.10%
Distance Actigraph Wrist H	110.04	52.20%	237.47	−17.39	60.50%	0.81	<0.001	49.24	23.30%
Distance Armband nH	127.26	60.40%	286.80	−32.28	75.70%	0.68	<0.001	65.92	31.30%
Distance Armband H	120.62	57.20%	288.25	−47.01	79.60%	0.72	<0.001	83.01	39.40%
Distance Pedometer Chest	27.20	12.90%	156.42	−102.02	61.30%	0.91	<0.001	61.67	29.20%
Distance Pedometer Hip	−20.51	−9.70%	28.68	−69.70	23.30%	0.98	<0.001	23.12	10.90%

## Supplementary Materials

Supplementary materials can be found at https://www.mdpi.com/1424-8220/19/11/2497/s1. Table S1: Characteristics of the selected devices.
