# Supplementary material for: Validity of the Walked Distance Estimated by Wearable Devices in Stroke Individuals
Source: Sensors (Basel). 2019 May 31;19(11):2497. doi: 10.3390/s19112497 (PMC6604102; doi:10.3390/s19112497)
Supplement: Supplementary file 1 [file sensors-19-02497-s001.pdf]

sensors

Supplementary Materials

Validity of the Walked Distance Estimated by Wearable Devices in Stroke Individuals

Maxence Compagnat [1,2,*], Charles Sebiyo Batcho [3,4], Romain David [2], Nicolas Vuillerme [5], Jean Yves Salle [1,2], Jean Christophe Daviet [1,2] and Stéphane Mandigout [1]

1 (Handicap, Aging, Autonomy, Environment) HAVAE EA6310, University of Limoges, city post code, country; JYVES.SALLE@chu-limoges.fr (J.Y.S.); jean-christophe.daviet@unilim.fr (J.C.D.); mandigout@unilim.fr (S.M.)
2 Department of Physical Medicine and Rehabilitation, University Hospital Center of Limoges, city post code, France; Romain-david@hotmail.fr
3 Center for Interdisciplinary Research in Rehabilitation and Social Integration (CIRRIS), Centre Intégré Universitaire de Santé et de Services Sociaux de la Capitale Nationale (CIUSSS-CN), city post code, QC, Canada; charles.batcho@fmed.ulaval.ca
4 Department of Rehabilitation, Faculty of Medicine, Université Laval, city post code, QC, Canada,
5 Department, University Grenoble Alpes, AGEIS, Grenoble post code,, France & Institut Universitaire de France, Paris post code, France; nicolas.vuillerme@univ-grenoble-alpes.fr

Table S1. Characteristics of the selected devices.

	Components	Recommended Placement	Measured Outcomes	Epoch	Cost
ActigraphGT3x[34]	3-axis accelerometer, digital filtering technology, integrated wear time and ambient light sensors	Wrist<br>Waist<br>Ankle<br>Thigh	Ambient Light Sensor<br>Heart Rate Monitoring<br>Proximity Detection<br>Wear Time Sensor<br>Daytime Activity Basic<br>Sleep Scoring<br>Inclinometer<br>Graphing<br>Data Vault Access	1/sec	335$
Sensewear ArmBand[30,40]	Multisensor array and biaxial accelerometer	Arm(Triceps Brachii)	Energy expenditure<br>Step count<br>Duration in postures	1/sec	900$
Pedometer[36]	Piezo electric	Around the neck<br>Hip	Step counts	Step by step	35$
